# Improvement of infection control services in Jeddah hospitals by program standardization and continuous auditing

**DOI:** 10.1186/1753-6561-5-S6-P275

**Published:** 2011-06-29

**Authors:** MA Halwani, NA Tashkandi, NY Nas

**Affiliations:** 1 The Administration of Infection Control, Health Affairs, Jeddah, Saudi Arabia

## Introduction / objectives

A proper infection control program in the Ministry of Health's hospitals-Jeddah, Saudi Arabia has never been applied based on focused and aimed scientific plans. The objective of this study was to monitor the enforcement of a scientifically based infection prevention and control program via continuous auditing.

## Methods

A standardization and continuous auditing program was implemented in the year 2009 in twelve hospitals. Three auditing visits were conducted throughout this year to estimate hospital-wide compliance of the required procedures and strategies using auditing sheets based on that year's target aims.

## Results

The first compliance rate for the first visit in the first year was 42% which increased to 66% during the second visit and to 78% during the third visit. During the first audit there were deficiencies in: general measures in infection control (43%), surveillance (31%), monitoring of infection control guidelines (81%), isolation practice (64%), infection control committee performance (66%) and education (46%). These deficiencies improved during the second audit as follows: (37%), (24%), (39%), (21%), (49%) and (25%) respectfully. During the third audit, deficiencies were as follows: (32%), (22%), (22%), (14%), (34%) and (9%) respectfully.

## Conclusion

A standardized infection control program for ongoing monitoring led to a significant improvement in infection control practices in all twelve hospitals in Jeddah. Consequently, these results might suggest that such a program can be applied to other Ministry of Health hospitals in the Kingdom.

## Disclosure of interest

None declared.

